# On-treatment decrease of NKG2D correlates to early emergence of clinically evident hepatocellular carcinoma after interferon-free therapy for chronic hepatitis C

**DOI:** 10.1371/journal.pone.0179096

**Published:** 2017-06-15

**Authors:** Po-sung Chu, Nobuhiro Nakamoto, Nobuhito Taniki, Keisuke Ojiro, Takeru Amiya, Yuko Makita, Hiroko Murata, Akihiro Yamaguchi, Shunsuke Shiba, Rei Miyake, Tadashi Katayama, Aya Ugamura, Akihiko Ikura, Karin Takeda, Hirotoshi Ebinuma, Hidetsugu Saito, Takanori Kanai

**Affiliations:** 1 Division of Gastroenterology and Hepatology, Department of Internal Medicine, Keio University School of Medicine, 35 Shinanomachi, Shinjuku-ku, Tokyo, Japan; 2 Center for Diagnostic and Therapeutic Endoscopy, Keio University School of Medicine, 35 Shinanomachi, Shinjuku-ku, Tokyo, Japan; 3 Kitasato University Kitasato Institute Hospital, 5-9-1 Shiragane-dai, Minato-ku, Tokyo, Japan; 4 Eiju Hospital, 2-23-16 Higashi-ueno, Ueno-ku, Tokyo, Japan; 5 International University of Health and Welfare Mita Hospital, 1-4-3 Mita, Minato-ku, Tokyo, Japan; 6 Division of Pharmacotherapeutics, Keio University School of Pharmacy, 1-5-30 Shibakoen, Minato-ku, Tokyo, Japan; National Taiwan University Hospital, TAIWAN

## Abstract

**Background and aims:**

Interferon (IFN)- free direct antiviral agents (DAAs) with rapid HCV eradication might evoke immunological reconstitutions, and some early recurrences of HCC after IFN-free DAAs have been reported. This study aimed to investigate whether natural killer group 2, member D (NKG2D) predicts early emergence of HCC after IFN-free DAAs.

**Methods:**

We conducted a clinical practice-based observational study of 101 patients infected with genotype 1 HCV who received IFN-free (DAAs), and stratified them into those who did or did not develop early (i.e., during the 6-month surveillance period following treatment.) recurrence or occurrence of clinically evident HCC. We also analyzed the peripheral blood mononuclear cells, both before treatment and at end of treatment (EOT), of 24 of the patients who received IFN-free DAAs, and 16 who received IFN-combined protease inhibitor.

**Results:**

We found early emergence of clinically evident HCC after IFN-free DAAs in 12 (12%) patients. Higher pre-treatment NKG2D expression, higher FIB-4 score, previous HCC history and failure to achieve sustained viral response were significant factors correlating to early HCC emergence. After IFN-free DAAs, a rapid decrease of NKG2D at EOT correlated with early HCC emergence in the IFN-free DAA-treated patients, but not in patients treated with the IFN-combined regimen. The decrease of NKG2D until EOT was predictive of early HCC emergence at a cut-off of -52% (AUC = 0.92).

**Conclusions:**

On-treatment decrease of NKG2D may be a useful predictor of early emerging HCC in patients treated with IFN-free DAAs.

## Introduction

Persistent hepatitis C virus (HCV) infection, which affects about 160 million people worldwide, is the major cause of liver cirrhosis and hepatocellular carcinoma (HCC). HCV-associated HCC, like some cancers in organs such as stomach or uterine cervix, reflects a model of infection-related carcinogenesis, in which chronic inflammation (i.e., the accumulation of immune cells) promotes tumor initiation and progression[1]. Recently, interferon (IFN)-free direct antiviral agents (DAAs) have been replacing pegylated-interferon (PEG-IFN)/ ribavirin (RBV) as a first-line treatment option recommended by international guidelines[2, 3], because of their high effectiveness and limited toxicity. Many studies have shown that sustained viral responses (SVRs) through IFN-combined therapies reduce liver-related complications[4, 5]. However, although IFN-free DAAs have been shown to eradicate HCV and improve liver residual function[6, 7], whether IFN-free DAAs can effectively prevent primary or secondary HCC is still unknown.

As a practical matter, patients who receive IFN-free DAAs are usually intolerant of IFN because of hematological toxicities that may be associated with advanced liver cirrhosis, old age, or decompensated liver functions. These are also risk factors for higher HCC incidence rates. Recently, several retrospective clinical studies of early recurrence of HCC after IFN-free DAAs suggested that robust immune re-constitution may be related to earlier HCC recurrence[8–10]; conversely, another study suggested this was not the case[11].

Several studies showed that IFN-free DAAs might evoke immune reconstitution of intrahepatic interferon-stimulated genes (ISGs)[12] and early responses in natural killer (NK) cells[13, 14]. However, these studies were based on pilot studies of clinical trials, and they were not tested clinically. How these immunological changes influence immunosurveillance of HCC is still not clear.

Here, we focused on natural killer group 2, member D (NKG2D), an activating receptor for MHC class I chain-related protein A/B (MICA/B) and other ligands. NKG2D is a widely studied immunoreceptor due to its major relevance in activating immune responses against both infected and transformed cells. NKG2D has been studied in the persistence of HCV infection[15] and HCV-associated HCC[16]. Peripheral blood NKG2D and its ligands have also been found to be dysregulated during the progression of various cancers[17, 18], and may be useful biomarkers[19, 20].

To find out how HCV eradication will influence NKG2D expression, and how this difference will correlate to early post-treatment HCC emergence, we conducted a real-world practice-based observational study of patients infected with genotype-1 HCV who were treated with IFN-free DAAs, and compared to those treated with IFN-combined regimen. We analyzed the correlation of NKG2D expression to early emergence of clinically evident HCC after treatment.

## Patients and methods

### Study subjects

**Fig 1:**
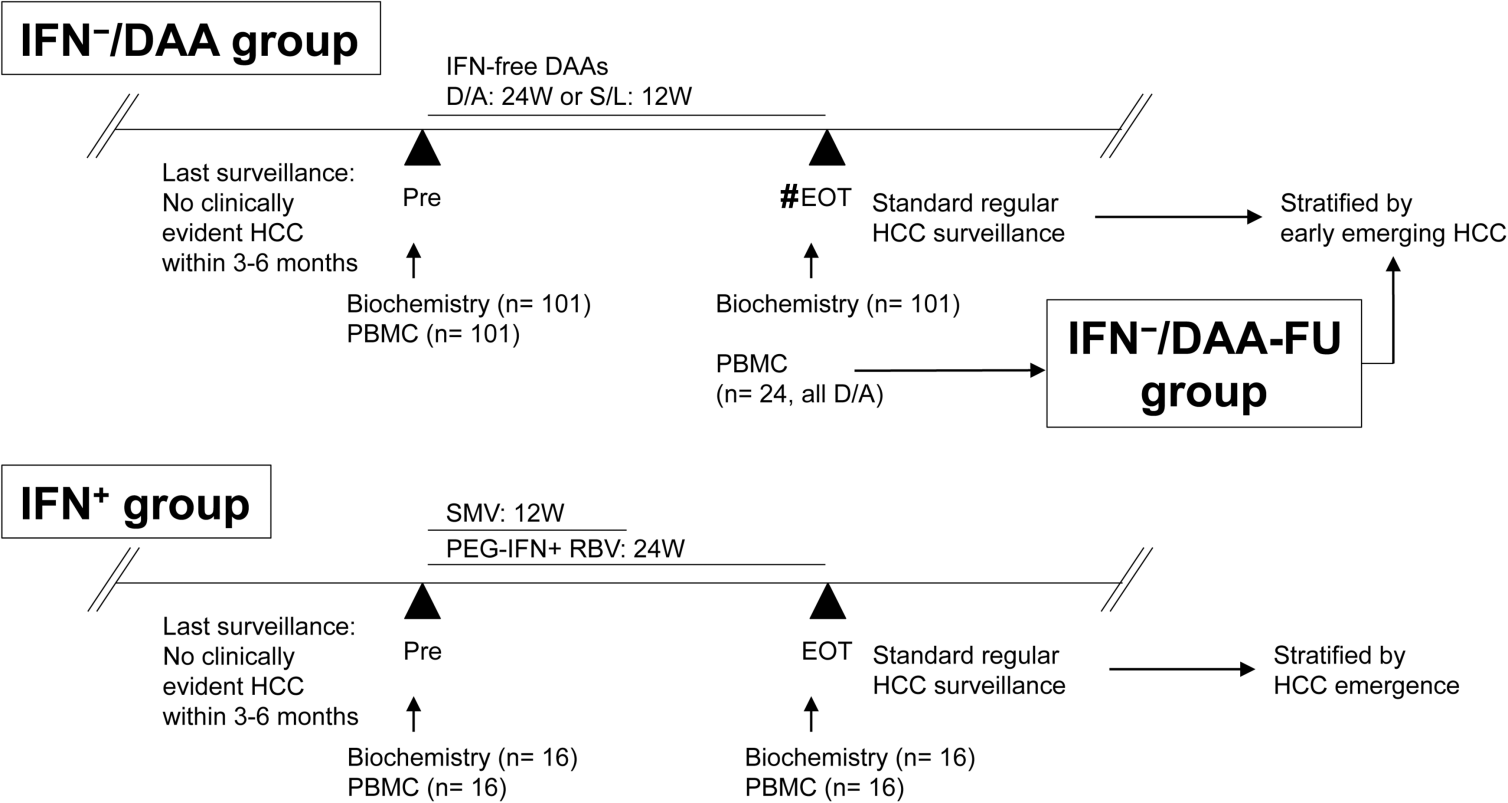
Scheme of this study. #: Samples were drawn at end of treatment (EOT) for most of the cases other than two cases that suffered from viral breakthrough (VBT). Abbreviations: IFN, interferon; DAAs, direct-acting anti-viral agents; D/A; daclatasvir and asuneprevir; S/L: sofosbuvir and ledipasvir; HCC, hepatocellular carcinoma; Pre, pre-treatment; EOT, end of treatment, PBMC, peripheral blood mononuclear cells; SMV, simeprevir; PEG, pegylated, RBV, ribavirin;.

This observational case-control study was specifically approved before we started, by the institutional review board of Keio University School of Medicine (No. 20140177), according to the guidelines of the 1975 Declaration of Helsinki (2008 revision). Recruited study subjects provided prior written informed consents, which included blood sampling, study participation and analysis of clinical data. All of the study subjects received standard of care and treatment, according to their clinical presentation. Measurement of viral kinetics was performed as previously described[15]. Negative plasma *HCV*-RNA at week 4 is defined as early viral response (EVR). Negative plasma *HCV*-RNA at 12 weeks after the end of treatment is considered to have a sustained virological response (SVR). We use RECICL2009[21], or its minor revision RECICL2015[22] accordingly during the study period, for evaluation of HCC treatment effect and response. Schema of the study groups is summarized in Fig 1.

IFN^−^/DAA group (the group that received IFN-free DAAs) included 101 IFN-free treatment-naïve patients with chronic HCV genotype-1 infections who had no evident HCC and were undergoing standard HCC surveillance by international recommendations[2] every 3–6 months. No patient of decompensated liver cirrhosis was included in this study. They were treated with daclatasvir (60mg once daily, DCV) and asunaprevir (100mg twice daily, ASV; both Bristol-Myers Squibb, New York, NY, USA) for 24 weeks, or sofosbuvir (400mg once daily, SOF) and ledipasvir (90mg once daily, LDV; both Gilead, Foster City, CA, USA) for 12 weeks. Their treatment started from November 2014 to June 2016. Biochemical studies were performed on sera from these patients retrieved on day 0 (pre-treatment; Pre) and on the end of treatment (EOT; 24 weeks for DAC/ASV and 12 weeks for SOF/LDV) for most cases who achieved SVR or for one case whose HCV-RNA relapsed, or at the time of viral breakthrough (VBT). All 101 patients had PBMCs retrieved on day 0 as part of their pre-treatment evaluations.

IFN^−^/DAA-FU (follow-up) group was a sub-group of IFN^−^/DAA group. Twenty-four patients who had been treated by DAC/ASV had additional PBMCs retrieved at the EOT. Cases of both IFN^−^/DAA group and IFN^−^/DAA-FU group were thereafter followed-up for HCC surveillance within 6 months after EOT, according to the guidelines[2]. They were stratified by emergence of HCC, including both recurrences and first occurrences. Any HCC recognized during this period was considered to be an early-emerging HCC. Clinical characteristics of IFN^−^/DAA group and IFN^−^/DAA-FU group are shown in Table 1 and Table 2, respectively. Liver function and tumor-related variables at 3 points in the study of the 12 patients who developed early-emerging HCC are summarized in S1 Table. Chronological flows of the 8 patients with recurrences of HCC, including tumor numbers, maximal sizes, BCLC stages, treatment, and follow-up image studies, are shown in S1 Fig. Comparison between IFN^−^/DAA group and IFN^−^/DAA-FU group is shown in S2 Table. Non-invasive fibrosis scores including aspartate aminotransferase (AST) to platelet ratio index (APRI, cut-off for METAVIR F4 cirrhosis as > 2.0), FIB-4 (cut-off for METAVIR F3-F4 advanced fibrosis as > 3.25), and Forns index (cut-off for METAVIR F0-F1 as < 4.2) were calculated as described before[23–25].

**Table 1:** Clinical characteristics of IFN^−^/DAA group, and comparison of cases stratified by early-emerging HCC.

Variables	Total	Post-treatment Early-emerging HCC		
		No	Yes	*P* (uni)
*n* (%)	101	89(88)	12(12)	–
Age, years	67 [24–85]	67 [24–85]	79 [50–82]	0.19
≧65 yrs, *n* (%)	62(62)	53(60)	9(75)	0.36
Sex (M: F), n (%)	38(38): 63(62)	35(37): 54(53)	3(25): 9(75)	0.53
Pre-treatment parameters				
ALT, IU/L	44[13–284]	44[13–284]	45[23–83]	0.97
AST, IU/L	49[19–246]	48[19–246]	73[20–129]	0.22
G-GTP, IU/L	31[11–268]	32[11–268]	30[18–79]	0.92
T-Bil, mg/dl	0.8[0.3–2.7]	0.8[0.3–2.7]	1.1[0.4–2.0]	0.0171*
Albumin, g/dl	4.3[3.3–4.9]	4.4[3–4.9]	3.8[3.3–4.3]	0.0002**
Platelet, 10^3^/μl	136[33–457]	145[33–457]	93[37–172]	0.007**
PT-INR	1.00[0.86–1.30]	1.00[0.86–1.30]	1.07[1.00–1.29]	0.0003**
4COL7s, ng/ml	6.7[3.4–17]	6.4[3.4–15]	9[5.1–17]	0.001**
AFP, ng/ml	6[1–685]	5[1–685]	17.5[3–171]	0.002*
*HCV*-RNA, LogIU/ml	6.3[3.5–7.5]	6.3[3.5–7.5]	6.0 [5.3–7.1]	0.25
Total cholesterol, mg/dl	159[107–241]	161[107–241]	133[110–184]	0.013*
Forns index	7.6[2.5–13]	7.5[2.5–13]	9.2[6.8–13]	0.001**
<4.2, *n* (%)	6(6)	6(7)	0(0)	1.00
FIB-4	3.87 [0.62–107]	3.58 [0.62–107]	10.14 [1.41–16.56]	0.002**
>3.25, *n* (%)	61(60)	50(56)	11(92)	0.03*
APRI	1.11[0.13–52]	1.00[0.13–52]	3.18[0.33–5.4]	0.007**
>2.0, *n* (%)	26(26)	18(20)	8(67)	0.002**
Previous HCC, *n* (%)	16(16)	8(9)	8(67)	<0.0001**
Previous IFN-combined, *n* (%)	43(43)	40(45)	3(25)	0.23
DAA type, *n* (%)				0.75
DCV/ASV	62(61)	55(62)	7(58)	
SOF/LDV	39(38)	34(38)	5(42)	
NKG2D, %	23.4 [1–71]	22 [1–53]	33[19–71]	0.006**
Post-treatment parameters				
SVR, *n* (%)	98(97)	88(99)	10(83)	0.04*

*, *P*< 0.05

**, *P*< 0.01.

Data are shown as median with the range within brackets. Abbreviations: 4COL7s: type 4 collagen 7s domain; AFP: alpha-fetoprotein; ASV: asunaprevir; CI: confidence interval; DAA: direct-acting agent; DCV: daclatasvir; F: female; G-GTP, gamma- glutamyl transpeptidase; HCC: hepatocellular carcinoma; HCV: hepatitis C virus; IFN: interferon; LDV: ledipasvir; M: male; SOF: sofosbuvir; SVR: sustained viral response; T-Bil, total bilirubin; uni: univariate.

**Table 2:** Clinical characteristics of IFN^−^/DAA-FU group, and comparison of cases stratified by early-emerging HCC.

Variables	Total	Early-emerging HCC		
		No	Yes	*P*
*N*	24	19	5	–
Age, years	67.5 [45–79]	67 [45–79]	72 [50–79]	0.39
Sex (M/F), n	10 /14	9/ 10	1/ 4	0.36
Pre-treatment parameters				
ALT, IU/L	58 [26–284]	59 [27–284]	57 [26–83]	0.89
AST, IU/L	67 [27–246]	61 [27–246]	76 [32–92]	0.72
G-GTP, IU/L	43 [19–268]	47 [24–268]	30 [19–79]	0.39
T-Bil, mg/dl	0.9 [0.4–2.7]	0.8 [0.4–2.7]	1.2 [0.7–1.8]	0.24
Albumin, g/dl	3.9 [3.3–4.8]	4.0 [3.3–4.8]	3.9 [3.3–4.1]	0.23
Platelet, 10^3^/μl	108 [47–221]	111 [47–221]	65 [49–94]	0.01*
4COL7s, ng/ml	7.9 [3.9–15]	7.5 [3.9–15]	8.1 [7.2–15]	0.26
AFP, ng/ml	12 [3–171]	9 [3–109]	23 [9–171]	0.14
*HCV*-RNA, LogIU/ml	6.3 [5.8–7.5]	6.4 [5.9–7.5]	5.9 [5.8–6.4]	0.02*
Total cholesterol, mg/dl	148 [107–230]	152 [107–230]	127 [120–173]	0.08
FIB-4	4.49 [1.21–25.80]	4.40 [1.21–25.80]	12.73 [4.34–16.56]	0.12
APRI	1.80[0.35–14.95]	1.49[0.35–14.95]	3.52 [0.97–4.43]	0.10
Previous HCC, *n* (%)	5 (21)	2 (11)	3 (60)	0.04*
Post-treatment parameters				
EVR, *n* (%)	20 (83)	16 (84)	4 (80)	1.00
SVR, *n* (%)	21 (88)	18 (95)	3 (60)	0.10
ALT, IU/L	21 [11–235]	17 [11–235]	29 [21–30]	0.24
AST, IU/L	28 [17–102]	25 [19–102]	33 [28–46]	0.07
G-GTP, IU/L	23 [12–150]	22 [12–150]	23 [15–28]	0.91
T-Bil, mg/dl	1.0 [0.5–2.0]	0.9 [0.5–1.4]	1.1 [1.0–2.0]	0.05
Albumin, g/dl	4.3 [3.7–4.9]	4.4 [3.7–4.9]	4.2 [4.1–4.5]	0.24
Platelet, 10^3^/μl	115 [54–202]	120 [54–202]	120 [59–125]	0.10
4COL7s, ng/ml	6.9 [4.3–12]	6.3 [4.3–12]	8.1 [7.5–9.2]	0.08
AFP, ng/ml	5 [3–591]	4 [3–9]	10 [4–591]	0.04*
Total cholesterol, mg/dl	166 [121–233]	167 [127–233]	143 [121–193]	0.08
FIB-4	3.06 [0.99–10.25]	2.73 [0.99–9.66]	5.92 [2.27–10.25]	0.08
APRI	0.64 [0.24–3.07]	0.61 [0.30–3.07]	1.15 [0.69–2.08]	0.04*
ΔNKG2D (NK cells), %	−42 [−80–+21]	−34 [−73–+21]	−57 [−80–−51]	0.005**

*, *P*< 0.05

**, *P*< 0.01.

Data are shown as median with the range within brackets. Abbreviations: 4COL7s: type 4 collagen 7s domain; AFP: alpha-fetoprotein; EVR: early viral response; F: female; HCC: hepatocellular carcinoma; HCV: hepatitis C virus; M: male; SVR: sustained viral response.

IFN^+^ group (the group received SMV/PEG-IFN/RBV triple therapy), a control group whose treatment started from June to October, 2014, included 16 patients with chronic HCV genotype-1 infection but no evident HCC, who underwent standard HCC surveillance by international recommendations[2] every 3–6 months. They were treated with a combination of weekly PEG-IFN-α2b, ribavirin (daily dose based on body weight; Schering-Plough KK, Tokyo, Japan) for 24 weeks, and simeprevir (150 mg once daily, SMV; Janssen KK, Tokyo, Japan) for the first 12 weeks. Serum biochemical studies and PBMCs were retrieved on day 0 (Pre) and on EOT (24 weeks). After EOT, these patients were followed-up for HCC surveillance every 3–6 months regularly, according to the guidelines[2]. Clinical characteristics that compare IFN^−^/DAA-FU group and IFN^+^ group are shown in S3 Table.

### Flow cytometry analysis

Patients’ PBMCs were isolated freshly without cryopreservation on the day of sampling, and were analyzed by flow cytometry as previously described[15]. Anti-human monoclonal antibodies, including those for CD3 (FITC, clone HIT3a, Becton-Dickinson [BD], Franklin Lake, NJ, USA), CD56 (PE-Cy7, clone B159, BD), CD4 (APC, clone Leu3a, BD), CD8 (APC-Cy7, clone SK1, BD), 7-amino-actinomycin D (7-AAD), NKG2D (PE, clone 1D11, BioLegend, San Diego, CA, USA), CD25 (APC-Cy7, clone M-A251, BD), and CD127 (PE, clone HIL-7R-M21, BD) were used. Dead cells were excluded from assessment by 7-AAD. Irrelevant anti-rat isotype antibodies (BD) were used to assess background fluorescence. Stained cells were analyzed using FACS Canto II (BD). Data were analyzed using FlowJo software (Tree Star Inc., Ashland, OR, USA). Gating strategies are shown in S2 Fig.

### Statistical analysis

Data were analyzed using JMP12 (SAS Institute, Inc. Cary, NC, USA), and are expressed as median with range or average± standard deviation (SD), as appropriate. Non-parametric Kruskal–Wallis tests were used to assess differences between groups. Categorical data were analyzed using Fisher’s exact test. To investigate the independent determining factors for early-emerging HCC, logistic regression models adjusted for covariates were generated. Receiver operating characteristic (ROC) analysis was performed to confirm the usefulness of various parameters to predict early HCC emergence. *P* < 0.05 was considered significant.

## Results

### Background characteristics: Significantly higher pre NKG2D correlates to early emerging of clinically evident HCC in IFN^−^/DAA group

In the IFN^−^/DAA group, 12 patients (12%) had early-emerging HCC (Table 1). Compared between patients with or without early-emerging HCC, pre-treatment parameters, including total bilirubin, serum albumin, platelet count, PT-INR, type 4 collagen domain 7s (4COL7s), FIB-4 score, APRI, Forns index, alpha-fetoprotein (AFP), total cholesterol, status of previous HCC history, SVR status, and total NKG2D expression differed significantly. Notably, 60% of the cases had advanced liver fibrosis (FIB-4 score >3.25), and 26% of them had liver cirrhosis (APRI >2.0). This reflected a fact that many patients who underwent IFN-free DAAs were ineligible for IFN-combined regimen due to advanced liver fibrosis. In multivariate analysis, the presence of pre-treatment higher total NKG2D expression, along with previous HCC history and non-SVR, was found to be significantly associated with early-emerging HCC, after adjustment for age, sex, AFP, FIB-4 score, and/or serum albumin (Table 3) in various models.

**Table 3:** Models of multivariate analysis in IFN-/DAA group predicting early- emerging HCC.

	Model 1			Model 2			Model 3		
Variables	HR	95%CI	*P*	HR	95%CI	*P*	HR	95%CI	*P*
HCC past history	62	7.4–1775	0.002**	24.1	3.8–302	0.003**	49.3	5.2–1700	0.006**
Non SVR	287	5.6–6.1x10^4^	0.01*	127	2.9–2.1x10^4^	0.02*	476	6.1–3740	0.02*
Pre NKG2D	285	3.5–1.1x10^8^	0.01*	72	1.9–3.4x10^6^	0.01*	840	5.6-x1.1x10^9^	0.01*
Pre FIB-4	1.3	1.1–1.6	0.02*	-	-	-	-	-	0.18
Pre Albumin	-	-	-	498	2.4–3.7x10^5^	0.04*	-	-	0.09

*, *P*< 0.05

**, *P*< 0.01.

Multivariate Model 1 was adjusted for “Age over 65 years,” “sex,” “pre-treatment alpha-fetoprotein,” “HCC past history,” “Non SVR,” and “pre-treatment FIB-4 scores.” Multivariate Model 2 was adjusted for all variables in Model 1 with “pre-treatment serum albumin,” exclusive of “pre-treatment FIB-4 score.”Multivariate Model 3 was adjusted for variables in Model 1 and “pre-treatment serum albumin.”“Age over 65 years,” “sex,” and “pre-treatment alpha-fetoprotein” are not significant variables in all Models. Abbreviations: HR, hazard ratio; CI, confidence interval.

**Fig 2:**
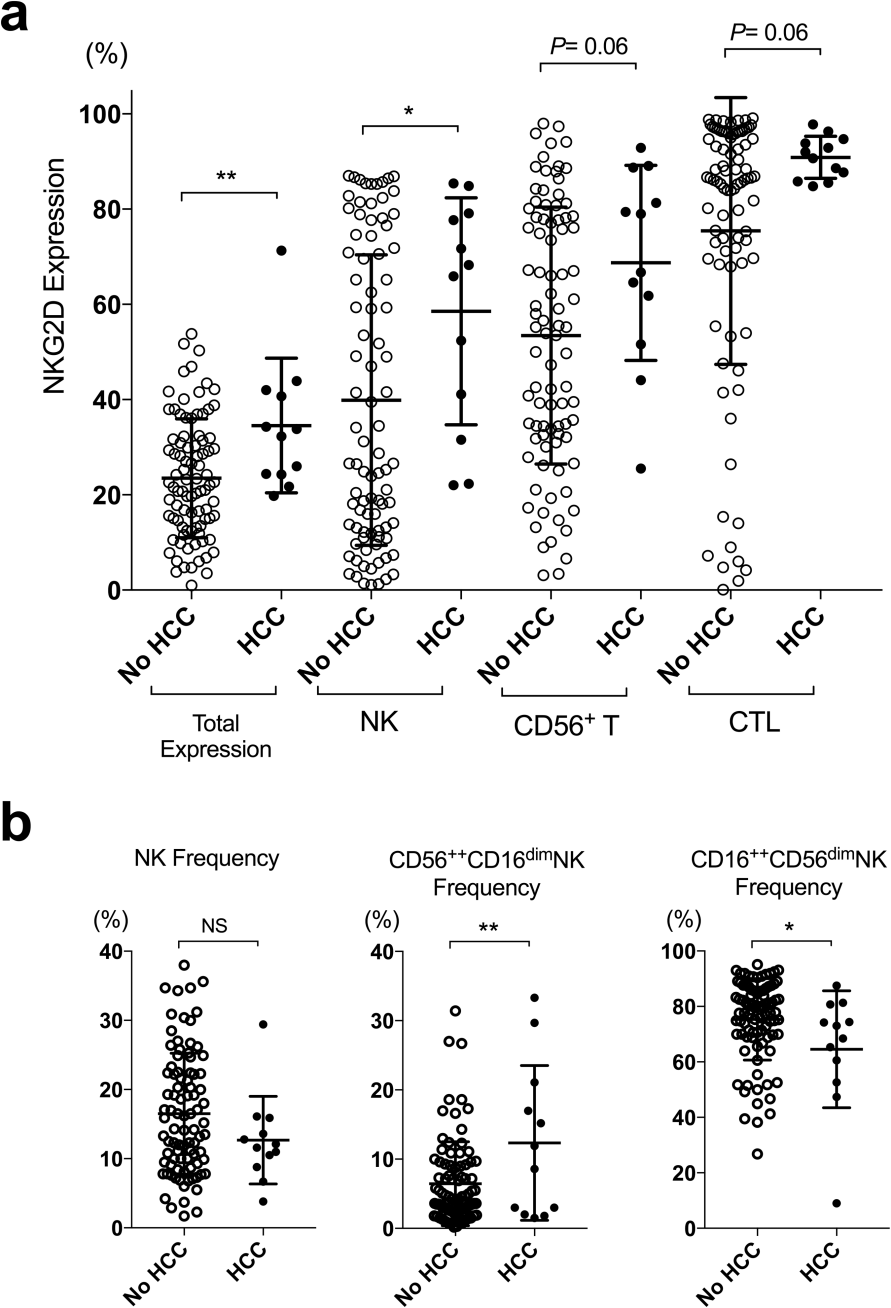
Pre-treatment NKG2D expression and frequencies of natural killer cell compartments stratified by early emergence of HCC or not (IFN^-^/DAA group). Filled circles represent cases with early emerging HCC and open circles represent cases without. Statistics were shown as mean with SD. *, *P*< 0.05; **, *P*< 0.01.

**Fig 3:**
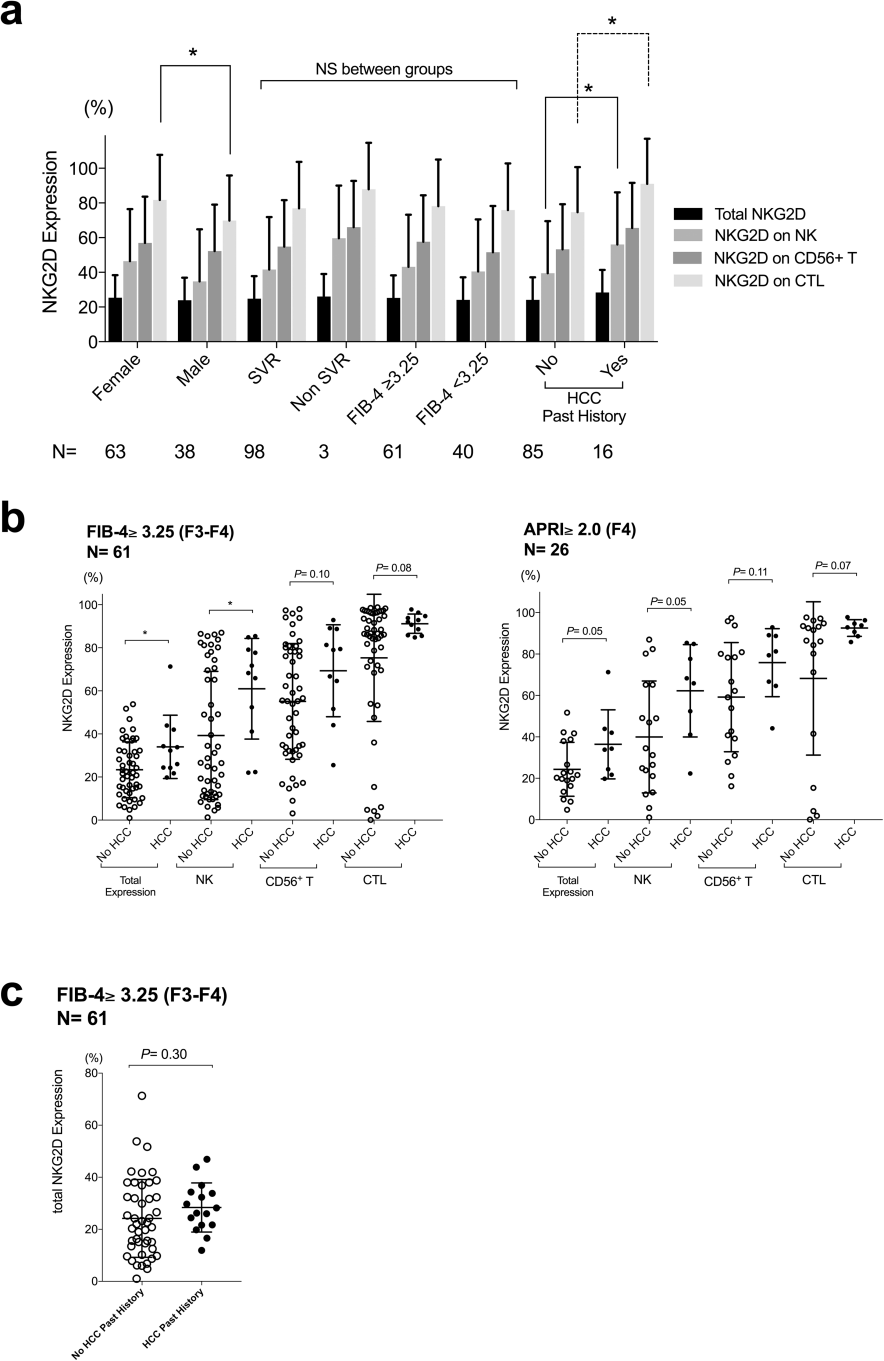
Background pre-treatment characteristics and NKG2D expression. Pre-treatment NKG2D expression was, (a) compared by sex, SVR status, FIB-4 score, and HCC past history with case numbers shown below each group; (b) compared by early emergence of HCC or not in cases restricted to advanced fibrosis (FIB-4 score over 3.25; left panel) and cirrhosis (APRI over 2.0; right panel); (c) compared between cases with or without HCC past history as cases restricted to advanced fibrosis (FIB-4 score over 3.25). Filled circles represent cases with early-emerging HCC and open circles represent cases without. Statistics were shown as mean with SD. *, *P*< 0.05; NS, not significant.

Pre-treatment peripheral blood NKG2D expression of total lymphoid cells and of individual type of immune cells, including NK cells, CD56^+^ T cells and cytotoxic T cells (CTLs), were shown in Fig 2A. Pre-treatment NKG2D expression of total lymphoid cells and of NK cells were significantly higher in cases that developed early HCC after IFN-free DAA treatment. Additionally, the pre-treatment frequency of CD56^++^CD16^dim^ NK cells was significantly higher, and that of CD16^++^CD56^dim^ NK cells was significantly lower in cases that developed early HCC after IFN-free DAAs (Fig 2B). Higher pre-treatment NKG2D expression on NK cells and CTLs correlated to previous HCC history, but did not correlated to SVR or FIB-4 scores (Fig 3A). As the study subjects were restricted to advanced fibrosis (FIB-4 score over 3.25) or cirrhosis (APRI over 2.0), the tendencies of higher pre-treatment NKG2D expression remained (Fig 3B), and the expression did not differ significantly when stratified by previous HCC history (Fig 3C). The analysis of the correlations of various biochemical studies and the NKG2D expression of individual immune cell types showed that higher pre-treatment NKG2D expression of NK cells correlated significantly to decreased liver protein synthesis, including a lower serum albumin levels or a more prolonged PT-INR (Table 4). Notably, NKG2D expression of NK cells, CD56^+^ T cells and CTLs all correlated slightly to serum AFP levels (Table 4).

**Table 4:** Various pre-treatment factors and their correlation to pre-treatment NKG2D expression.

*Pre-treatment Variables*		Total NKG2D	NKG2D on NK	NKG2D on CD56+ T	NKG2D on CTLs
Age	R	0.04	0.06	0.1	0.18
	*P*	0.69	0.56	0.35	0.07
AST	R	0.14	0.09	0.17	-0.15
	*P*	0.17	0.36	0.08	0.12
T-Bil	R	0.09	0.11	0.06	0.03
	*P*	0.37	0.25	0.49	0.77
Albumin	R	-0.03	-0.25	-0.26	-0.08
	*P*	0.34	0.01*	<0.01**	0.49
PI-INR	R	0.17	0.23	0.17	0.07
	*P*	0.10	0.03*	0.09	0.48
Total cholesterol	R	0.03	0.06	0.09	0.09
	*P*	0.73	0.52	0.37	0.36
Platelet	R	-0.20	-0.08	-0.03	0.00
	*P*	0.04*	0.42	0.33	0.98
4COL7s	R	0.11	0.12	0.20	-0.02
	*P*	0.29	0.22	0.05	0.81
*HCV*-RNA	R	-0.08	-0.10	-0.03	-0.04
	*P*	0.43	0.31	0.71	0.68
AFP	R	0.30	0.21	0.23	-0.24
	*P*	<0.01**	0.04*	0.02*	0.01*

*, *P*< 0.05

**, *P*< 0.01.

Abbreviations: 4COL7s: type 4 collagen 7s domain; AFP: alpha-fetoprotein; G-GTP, gamma- glutamyl transpeptidase; HCV: hepatitis C virus; R, correlation coefficient; T-Bil, total bilirubin.

These results accord with previous studies which showed that background liver fibrosis status might greatly influence HCC development, in both untreated HCV patients[26] and SVR patients treated by PEG-IFN/RBV[27]. With standard surveillance according to the guidelines, nine out of twelve (75%) of the early-emerging HCCs in this current study were of early-stage diseases (BCLC 0-A), and most of the lesions could be ideally controlled, as shown in S1 Table and S1 Fig.

### NKG2D expression decreased significantly at EOT for patients on IFN-free DAAs

In most cases of IFN^−^/DAA group, peripheral blood *HCV*-RNA decreased below 1.2 Log IU/ml soon after treatment with IFN-free DAAs (S3A Fig). The pre-treatment and EOT biochemical studies showed that IFN-free DAA administration significantly normalized AST and ALT, regardless of whether patients developed early-emerging HCC or not (S3B Fig).

Because HCV is thought to be noncytopathic[28], rapid AST/ALT normalization might be a result of rapid depression, whether quantitatively or functionally, of intrahepatic cytotoxic inflammatory cells.

**Fig 4:**
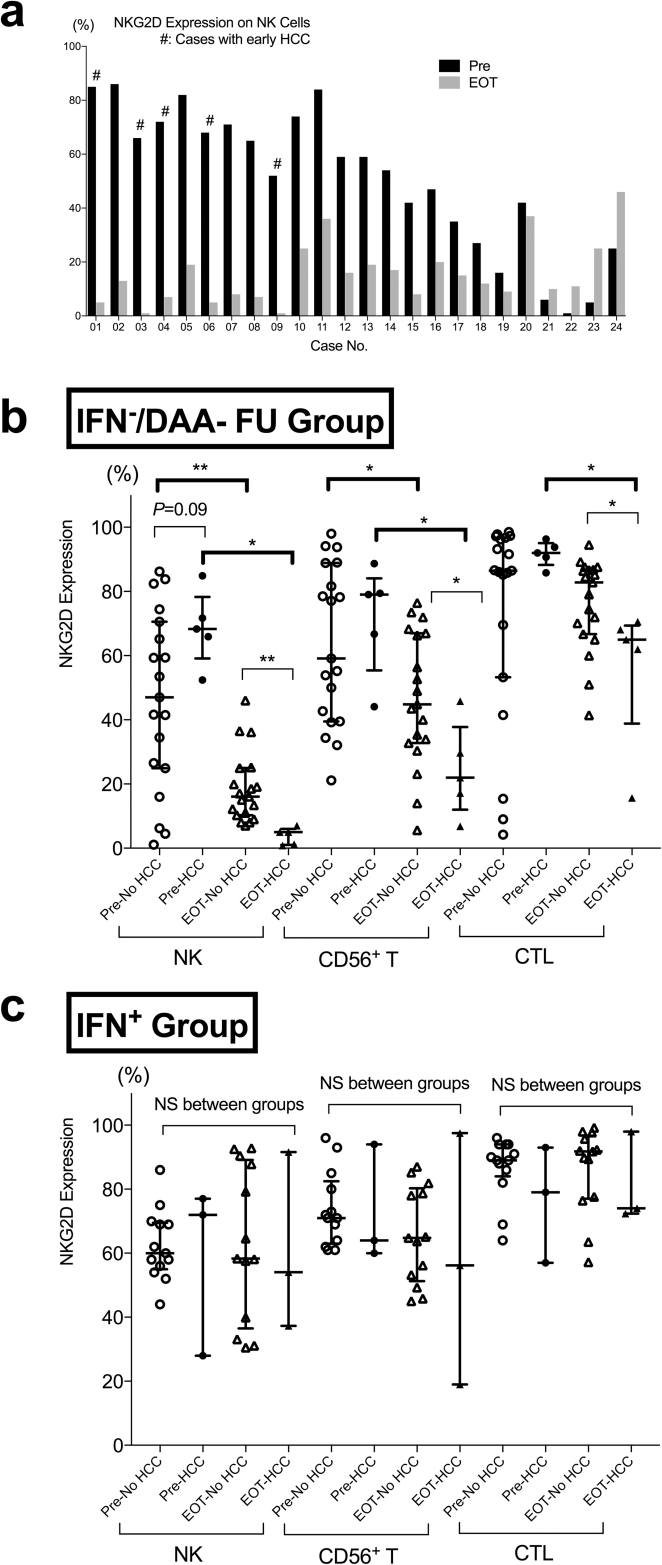
Pre- and End of Treatment NKG2D Expression of NK cells in IFN^−^/DAA-FU or IFN^+^ group. (a) Pre and EOT NKG2D expression of NK cells in each cases of IFN^−^/DAA-FU group. # represents cases with early-emerging HCC. (b, c) NKG2D Expression of NK cells in IFN^−^/DAA-FU group and in IFN^+^ group were compared between cases with or without early emergence of clinically evident HCC. Filled circles (pre) or triangles (EOT), cases with HCC; open circles (pre) or triangles (EOT), cases without HCC. Statistics were shown as median with interquartile ranges. *, *P*< 0.05; **, *P*< 0.01; NS, not significant.

Patient background characteristics of the IFN^−^/DAA-FU group were shown in Table 2. The factors that were significantly correlating to early HCC emergence were similar to those in the IFN^−^/DAA group (Table 1). As shown in Fig 4A, in IFN^−^/DAA-FU group, NKG2D expression on NK cells was significantly decreased generally from pre-treatment to EOT. NKG2D expression of NK cells decreased more significantly (18.6% vs 4.5%, *P* = 0.003, Fig 4B), in cases that developed early HCC. NKG2D expression of CD56^+^ T cells and CTLs had the same trend of changes (Fig 4B). On the other hand, the frequency of CD56^++^CD16^dim^ NK cells tended to (*P* = 0.11) decrease in cases without early HCC, but stayed relatively the same in cases with early HCC. There were no significant changes in frequencies of NK cells and the CD16^++^CD56^dim^ compartment (S4 Fig). Contrarily, in IFN^+^ group, NKG2D expression of NK cells remained relatively unchanged, without significant differences and stayed almost the same (Fig 4C), even for 12 to 24 weeks after EOT (S6A Fig).

In order to clarify the contribution of high levels of pre-NKG2D and the decreased levels of EOT-NKG2D, we did a linear regression analysis between “ΔNKG2D,” “pre-treatment NKG2D,” and “EOT NKG2D” on NK cells. Pre-treatment NKG2D highly correlated to ΔNKG2D (S5A Fig), however, EOT-NKG2D also significantly correlated to ΔNKG2D (S5A Fig). Therefore, significant “down-regulation,” as shown in Fig 4B, rather than merely “normalization,” of the NKG2D expression levels, contributed to ΔNKG2D. Furthermore, pre-NKG2D showed no significant correlations to EOT-NKG2D (S5B Fig).

### An increased compartment of CD25^high^ helper T cells correlated to NKG2D decrease on NK cells After IFN-free DAA

**Fig 5:**
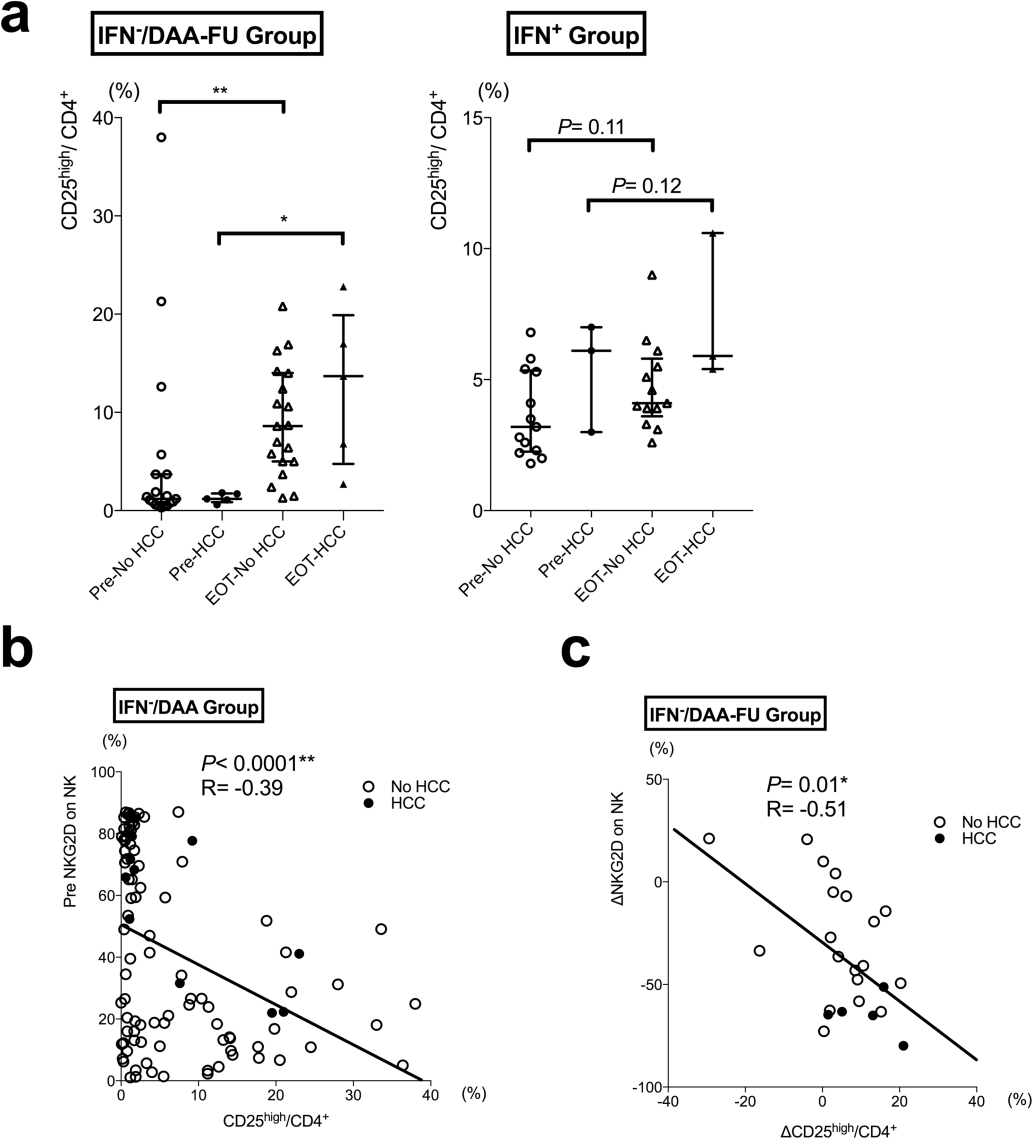
CD25^high^ helper T cells and the correlation to NKG2D expression of NK cells. (a) CD25^high^ helper T cells in IFN^−^/DAA-FU group and IFN^+^ group were compared between cases with or without early emerging HCC. (b) Correlations between pre-treatment NKG2D expression of NK cells and the compartment of CD25^high^ helper T cells in IFN^−^/DAA group. (c) Correlations between pre- and end of treatment changes in NKG2D expressions (ΔNKG2D) of NK cells and the changes in the compartment of CD25^high^ helper T cells (ΔCD25^high^/CD4^+^), in IFN^−^/DAA-FU group. Filled circles (pre) or triangles (EOT), cases with HCC; open circles (pre) or triangles (EOT), cases without HCC. Statistics were shown as median with interquartile ranges. *, *P*< 0.05; **, *P*< 0.01.

Since increased intrahepatic TGF-β has been reported in HCV-related liver fibrosis[29], we further focused our analysis on the influence of IFN-free DAA treatment on CD25-expressing helper T cells (defined as CD25^high^CD127^−^CD4^+^ T cells, suggested to be a major source of TGF-β[30]). In the IFN^−^/DAA group, the compartment of CD25^high^ helper T cells significantly elevated in both the non-HCC group and the HCC group (Fig 5A). Furthermore, when we looked on correlations between NKG2D expression of NK cells and the compartment of CD25^high^ helper T cells, pre-treatment NKG2D expression on NK cells significantly inversely correlated to the compartment of CD25^high^ helper T cells (Fig 5B). We also noticed that the pre-treatment and EOT differences in NKG2D expression (ΔNKG2D) on NK cells significantly correlated to the differences of CD25^high^ helper T cells (ΔCD25^high^/CD4^+^), in IFN^−^/DAA group (Fig 5C), but not in IFN^+^ group (S6B Fig).

### On-treatment decreased of NKG2D correlated to early-emerging HCC

Of the 12 patients with early-emerging HCC in this study, ten (83%) showed reduced or stably low levels of serum AFP (S1 Table). Serum AFP is widely used to help predict post-SVR HCC emergence after IFN-combined therapies[31]. However, as serum AFP is also related to the altered hepatocyte architectures in CHC[32], rapid normalization of serum AFP after IFN-free DAAs could be misleading; cautious re-evaluation of this useful clinical tumor marker is needed.

**Fig 6:**
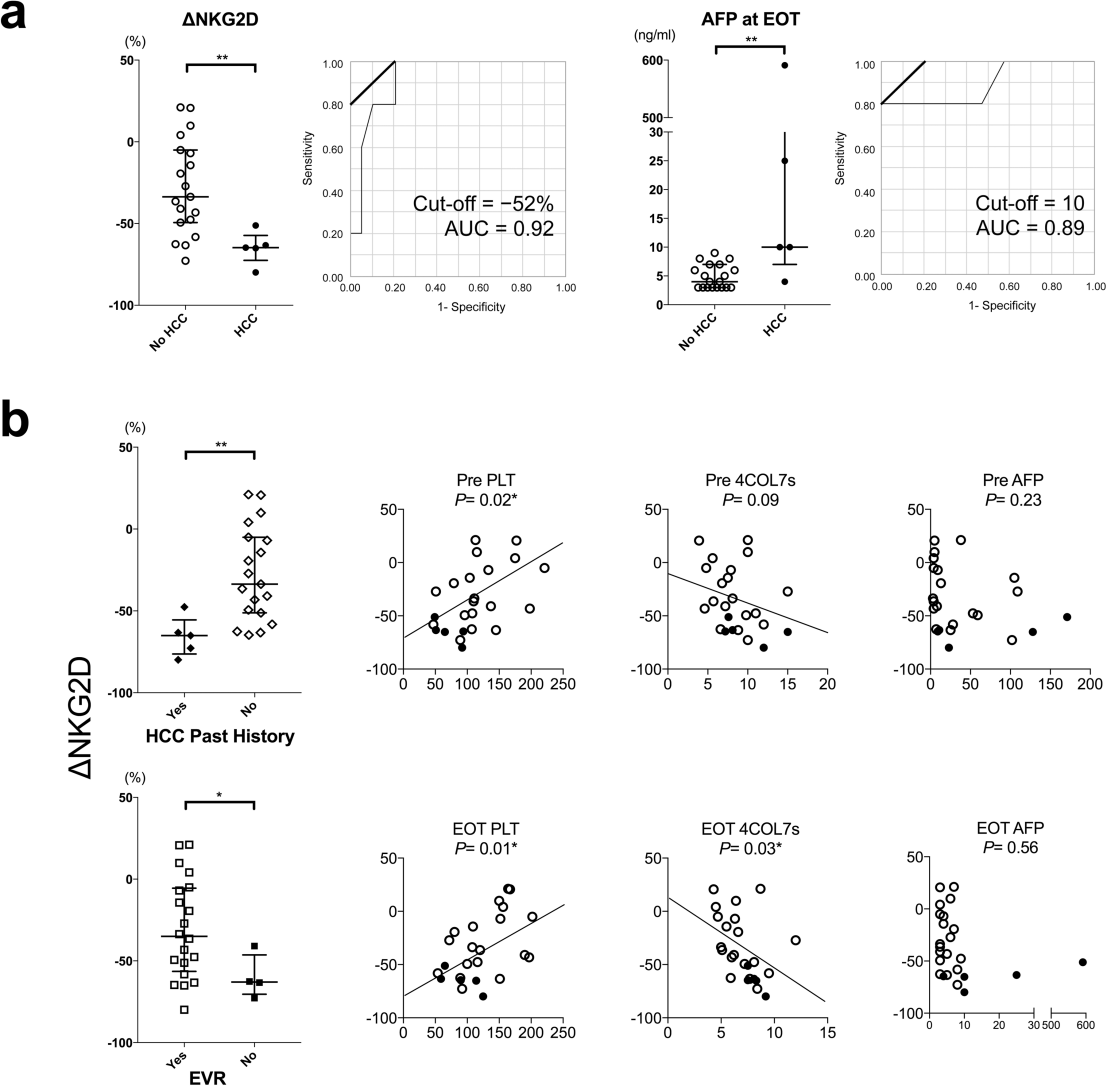
On-treatment decreased of NKG2D correlated to early-emerging HCC. (a) Changes of NKG2D expression from pre to EOT (ΔNKG2D) were compared in the IFN^−^/DAA-FU group. The ROC analysis of ΔNKG2D and AFP at EOT as a predictive factor to early HCC emergence were shown. (B) Correlations of ΔNKG2D to other background characteristics and biochemical factors in the IFN^−^/DAA-FU group were shown. Y-axis: ΔNKG2D expression on NK cells. Filled circles (pre) or triangles (EOT), cases with HCC; open circles (pre) or triangles (EOT), cases without HCC. Statistics were shown as median with interquartile ranges. *, *P*< 0.05; **, *P*< 0.01.

Because we noticed a significantly greater decrease in NKG2D expression of NK cells from pre-treatment to EOT (ΔNKG2D) in patients with early-emerging HCC (Fig 4B), we conducted ROC analysis of IFN^−^/DAA-FU group if ΔNKG2D predicted early-emerging HCC. The analysis showed that a cut-off of 52% decrease or more apparently predicted early-emerging HCC with an AUC = 0.92 in this model. Additionally, serum AFP at EOT had an AUC = 0.89 in the ROC analysis of IFN^−^/DAA-FU group at a cut-off value of 10ng/ml (Fig 6A). A more profound decrease of on-treatment NKG2D correlated to past history of HCC, EVR, and platelet count before treatment and at EOT, but didn’t correlate to AFP levels before treatment or at EOT (Fig 6B). On-treatment dynamics NKG2D might be a useful predictor of early-emerging HCC after IFN-free DAAs.

## Discussions

To our knowledge, this is the first real-world practice-based study to focus on immunological changes that correlate with early HCC emergence, with regard to IFN-free vs. IFN-combined regimens. Pre-treatment higher NKG2D expression and more profound NKG2D decrease on-treatment were noticed in patients with early-emerging HCC after IFN-free DAAs.

Previous studies[8–11] discussed the possibility of clinically undetectable HCCs or mistaken initial staging before administering IFN-free DAAs. In cirrhotic livers, transforming cells with genetic damages might have existed before they become transformed and large enough to be clinically detectable[33]. Additionally, subclinical HCCs could be cleared by host immunosurveillance in an early stage[34] before they become evident. Golden-Mason et al. demonstrated increased hepatic interleukin-15 (IL-15) promotes intrahepatic NK homeostasis, in chronic HCV infection[35]. IL-15 has also been showed to promote MICA/B- NKG2D interaction in CHC[36]. Therefore, higher pre-treatment levels of NKG2D (as shown in Table 1 and Fig 2) could result from such imbalance of inflammatory cytokines from both immune cells or other hepatic non-parenchymal cells such as hepatic stellate cells, especially in highly fibrotic livers, like the cases in IFN^−^/DAA group of this current study, even if no HCC was clinically evident at the time point of start of IFN-free DAA administration. Recently, Prenner et al. showed in a retrospective study that the existence of active HCC might reduce the rate to achieve SVR by IFN-free DAAs[37]. As we also showed in Table 1 that non-SVR correlated to early emergence of clinically evident HCC, pre-treatment existence of clinically undetectable HCC may be suggested as a causative factor for treatment failure.

Natural killer cell can be classified as immunoregulatory (cytokine-productive) CD56^++^CD16^dim^ and cytotoxic CD16^++^CD56^dim^ compartments[38]. Previous reports have showed an increased immunoregulatory CD56^++^CD16^dim^ compartment[39] and a decreased cytotoxic CD16^++^CD56^dim^ compartment[40] in chronic hepatitis C. To our knowledge, this current study is the first to demonstrate the significant differences of the two compartments between cases that developed HCC after treatment or not (Fig 2B).

In this study, various possible mechanisms might explain why on-treatment immune cells behave differently than in those undergoing IFN-free DAAs and IFN-combined therapy. First, the external administration of IFN-α might play a role. Reportedly, ISGs can be greatly decreased in patients treated with IFN-free DAAs[12], thus external IFN might explain why NKG2D expression on NK cells is maintained in IFN-combined therapy. Stegmann et al. showed that ex vivo stimulation of NK cells by IFN-α helped increase recognition of HCV-infected hepatoma cells[41]. Second, NKG2D ligands have been implicated in immune escape of tumor cells[42] and viral-induced HCC[16]. Mechanisms such as increased soluble NKG2D ligand shedding from HCC[43] could be possible, and should be further studied. Third, an increased compartment of CD25^high^ helper T cells, as shown in the current study, may down-modulate NKG2D-expressing NK cells through TGF-β[44–46]. Recently, Langhans et al. reported that peripheral FOXP3^+^CD25^+^CD4^+^ regulatory T cells may increase persistently after successful DAA treatment[47]. However, whether and how TGF-β changes during the rapid eradication of HCV by IFN-free DAAs still need to be investigated.

In earlier clinical trial-based PBMC studies, Spaan et al. demonstrated, in 12 patients who received DCV/ASV, a significant decrease of tumor necrosis factor-related apoptosis-inducing ligand (TRAIL) expression on NK cells from day 0 to 12W—i.e., half of the treatment course[14]. Serti et al. showed a rapid decrease in viremia and levels of inflammatory cytokines were associated with decreased activation of intrahepatic and blood NK cells in 13 patients who received DCV/ASV[13]. However, these studies showed relatively no significant change in NKG2D expression as we show here. The discrepancies in NKG2D expression between studies in chronic HCV infection are thought to reflect background host factors, such as degree of background fibrosis, or possible clinically undetectable pre-cancerous lesions in this real-world practice setting. In this current study, pre-treatment fibrotic status of the liver, implicated as higher pre-treatment FIB-4 scores (Tables 1 and 3), decreased hepatic protein synthesis (Table 4), and lower platelet counts (Fig 6B), were suggested to correlate to the differences in NKG2D expression.

Decreased NKG2D (Fig 4) and increased compartment of CD25^high^ helper T cells (Fig 5) are generally thought to be unfavorable to immunosurveillance of cancer cells[45]; these events might demonstrate how the influence on host immune response when a persistent infectious factor (in this case, HCV) is rigorously eradicated. Our study here suggested that rapid viral suppression by IFN-free DAAs may depress cytotoxic inflammatory cells, thus leading to a state of temporal relative immunosuppression due to immune reconstitution, not only to transformed cells as shown in this study, but also to cells co-infected with viruses such as hepatitis B virus (HBV) or herpes viruses, as increased reactivations of these viruses have been reported in patients who undergo IFN-free DAA treatment[48, 49]. However, in this study, successful HCV eradication (leading to SVR or not) was still an optimistic predictor of HCC development (Table 3). As with SVR after IFN-combined therapies that were effective for a second recurrence of HCC, but not for the first[50], we must still manage HCCs that may have existed before administration of IFN-free DAAs, even if they are not clinically evident. As IFN-free DAAs effectively lead to viral eradication, we might use IFN-free DAAs, if we are careful, in patients who are suspected to be at higher HCC risk, such as those with advanced fibrosis, or past history of HCC. Additionally, because immune cells might react quite differently to treatments with or without external IFN-α, what we have learned from previous IFN-combined therapies, including post-treatment follow-up parameters, intervals or durations, might also need to be re-evaluated.

A major limitation of this study is its observational design that is exploratory in nature. Subsequent studies with larger subject numbers will be needed to solidify the associations described in this study. We cannot conclude if there is a causal relationship between DAA administration, the on-treatment NKG2D decease, and HCC emergence. Again, we have to emphasize that this current study alone is not enough for indicating whether IFN-free DAAs increase the emergence of HCC or not. Additionally, due to IFN tolerability, background characteristics vary between study cohort (IFN^-^/DAA group) and the control (IFN^+^ group) in liver fibrotic status (S3 Table). Besides external IFN, the less fibrotic liver may also contribute to the maintenance of NKG2D expression in IFN^+^ group. Because only four cases with *de novo* early HCC were included in this study, our current data along may not be sufficient to conclude if there is any difference compared with early recurrence.

In conclusion, In this study based on real-world clinical practice, we found that early emergence of clinically evident HCC after IFN-free DAA treatment correlated to significant and profound decrease of NKG2D expression on NK cells, compared with that of patients who were treated with IFN-combined therapy. Stricter monitoring and use of novel non-invasive markers to predict fibrosis or HCC development are warranted. On-treatment dynamics of peripheral blood NKG2D expression could be a useful predictor of HCC in patients receiving IFN-free DAAs.

## Supporting information

S1 Fig Chronological flow of 8 patients with early-recurring HCC (i.e., who previously had HCC). The start of each case shows the patient’s last established active HCC before receiving IFN-free DAAs. Gd-EOB-DTPA, gadolinium ethoxybenzyl diethylene triamine pentaacetic acid; RFA, radiofrequency ablation; TACE, trans-arterial chemoembolization. (For other abbreviations, please see the main text.).
(TIFF)

S2 Fig Gating strategies. (TIFF)

S3 Fig Viral dynamics (a) and biochemial changes (b) stratified by early HCC emergence in IFN^−^/DAA group.
Statistics were shown as mean with SD. *, *P*< 0.05; **, *P*< 0.01. Units: HCV-RNA: LogIU/ml; AST: IU/L; ALT: IU/L; AFP: ng/ml. VBT, viral breakthrough.
(TIFF)

S4 Fig Changes of frequencies of NK cells and their compartments after IFN-free DAAs. Statistics were shown as mean with SD. *, *P*< 0.05; **, *P*< 0.01. NS, not significant.
(TIFF)

S5 Fig Correlations between ΔNKG2D, pre-treatment NKG2D and EOT NKG2D expressions on NK cells in IFN^-^/DAA-FU group. (a) Linear regression analyses of ΔNKG2D and pre- or EOT NKG2D expressions. (b) Linear regression analyses of pre- and EOT NKG2D expressions. Filled circles represent cases with early emerging HCC and open circles represent cases without. R, correlation coefficient; *, *P*< 0.05; **, *P*< 0.01.
(TIFF)

S6 Fig NKG2D expression of NK cells in IFN^+^ group at pre-treatment, end of treatment (EOT), and 12–24 weeks post-treatment. (TIFF)

S1 Table Liver function and tumor-related variables of patients with early emerging HCC (recurrence/occurrence) at three time-points of the study. CPT, Child-Pugh-Turcotte scores; PS, performance status; R/O, recurrence or occurrence; RFA, radiofrequency ablation. TACE, trans-arterial chemoembolization. (For other abbreviations, please see the main text.).
(DOCX)

S2 Table Clinical characteristics of IFN^-^/DAA and IFN^-^/DAA-FU groups. (DOCX)

S3 Table Clinical characteristics of IFN^-^/DAA-FU and IFN^+^ groups. (DOCX)

## Abbreviations

4COL7s: 7s domain of type 4 collagen
AFP: alpha-fetoprotein
ALT: alanine aminotransferase
AST: aspartate aminotransferase
ASV: asunaprevir
BCLC: Barcelona Clinic Liver Cancer
CHC: chronic hepatitis C
CTL: cytotoxic T cells
DAA: direct antiviral agent
DCV: daclatasvir
EOT: end-of-treatment
HCC: hepatocellular carcinoma
HCV: hepatitis C virus
IFN: interferon
ISG: interferon-stimulated gene
LDV: ledipasvir
MICA: MHC class I chain-related protein A
NK: natural killer
NKG2D: natural killer group 2, member D
PBMC: peripheral blood mononuclear cell
PEG: pegylated
RBV: ribavirin
SMV: simeprevir
SOF: sofosbuvir
SVR: sustained viral response
TGF: transforming growth factor
Treg: regulatory T cell
VBT: viral breakthrough
