# Supplementary figures and images for: On-treatment decrease of NKG2D correlates to early emergence of clinically evident hepatocellular carcinoma after interferon-free therapy for chronic hepatitis C
Source: PLoS One. 2017 Jun 15;12(6):e0179096. doi: 10.1371/journal.pone.0179096 (PMC5472371; doi:10.1371/journal.pone.0179096)

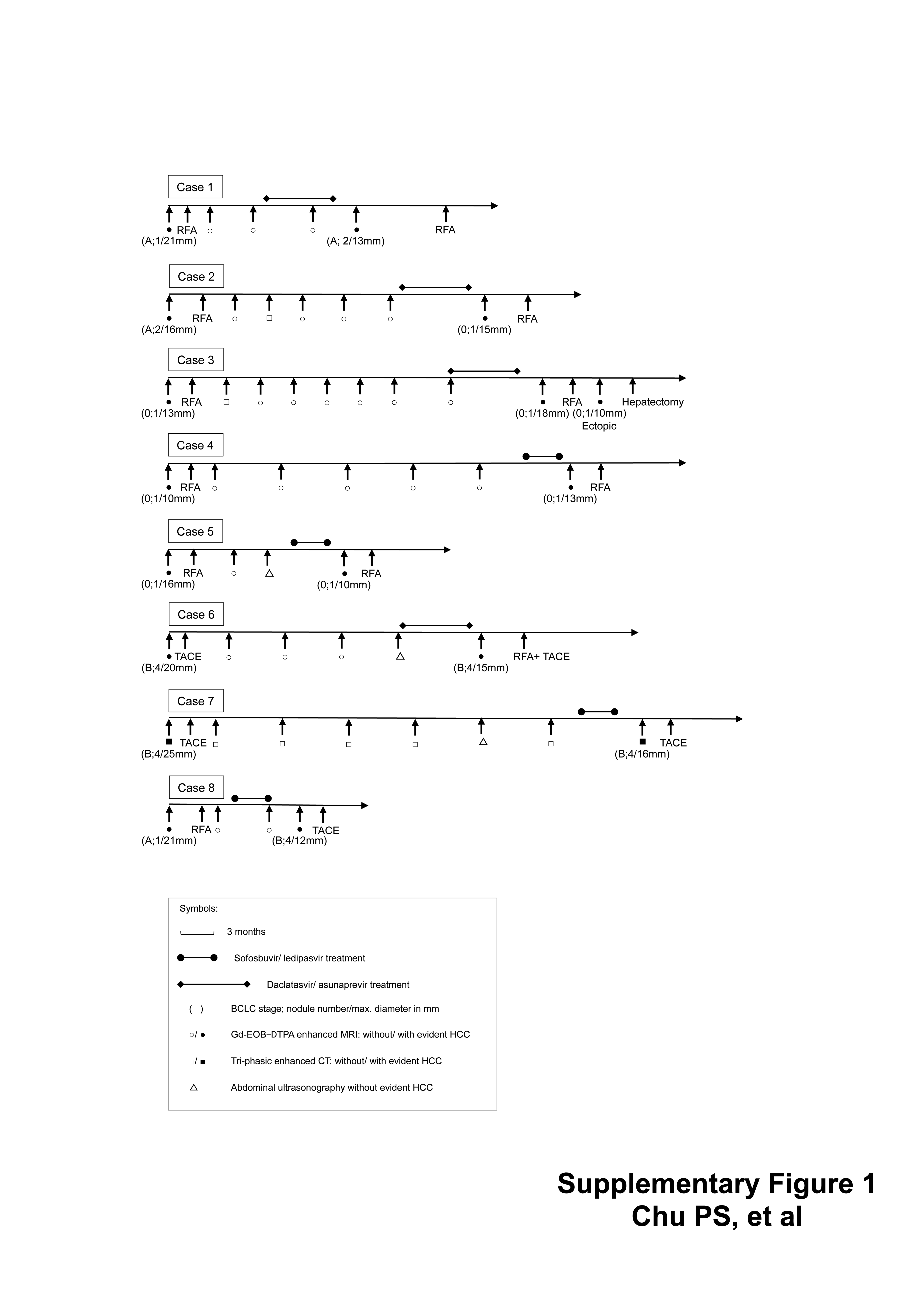

Supplement: S1 Fig — The start of each case shows the patient’s last established active HCC before receiving IFN-free DAAs. Gd-EOB-DTPA, gadolinium ethoxybenzyl diethylene triamine pentaacetic acid; RFA, radiofrequency ablation; TACE, trans-arterial chemoembolization. (For other abbreviations, please see the main text.). (TIFF) [file pone.0179096.s001.tiff]

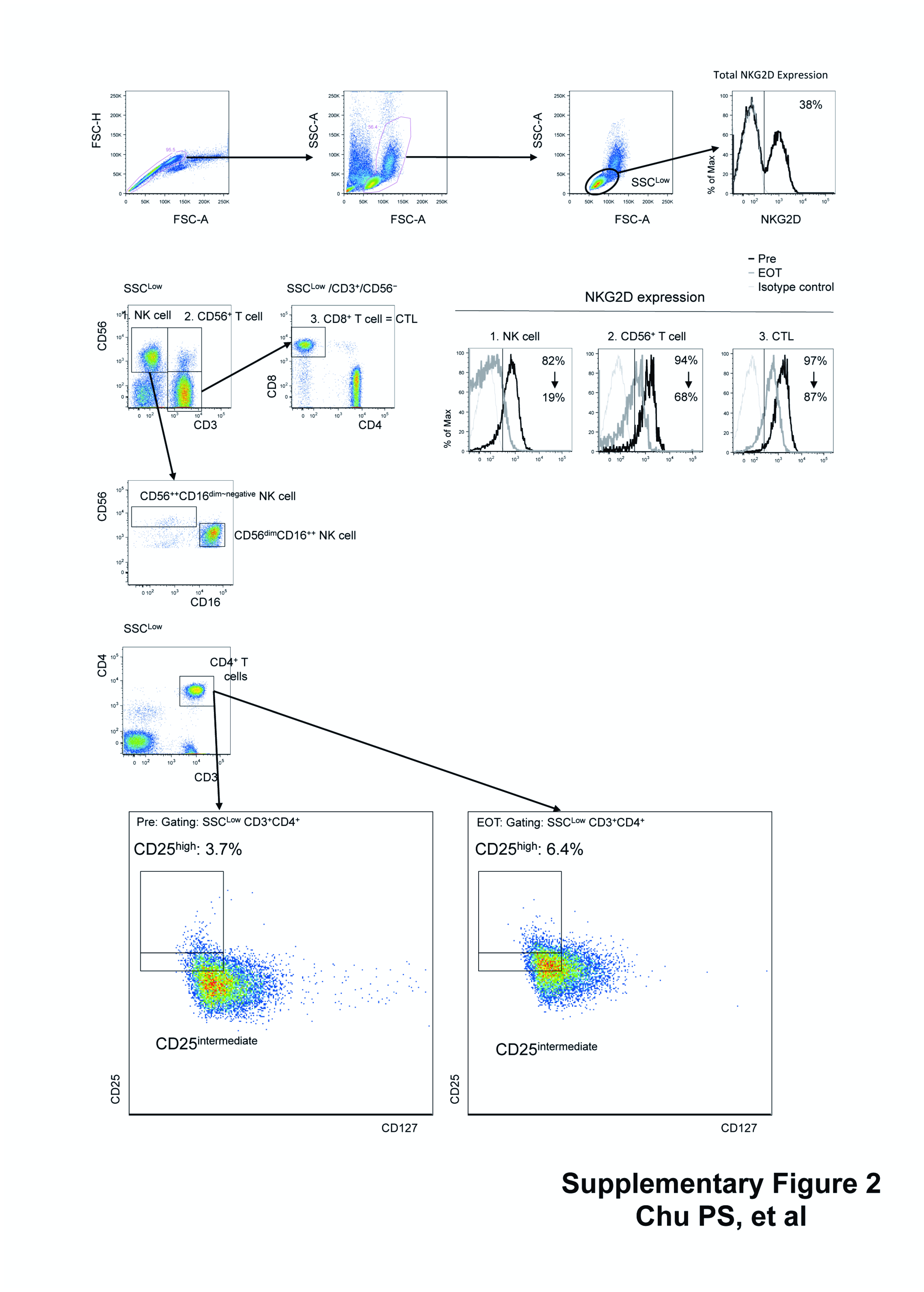

Supplement: S2 Fig — (TIFF) [file pone.0179096.s002.tiff]

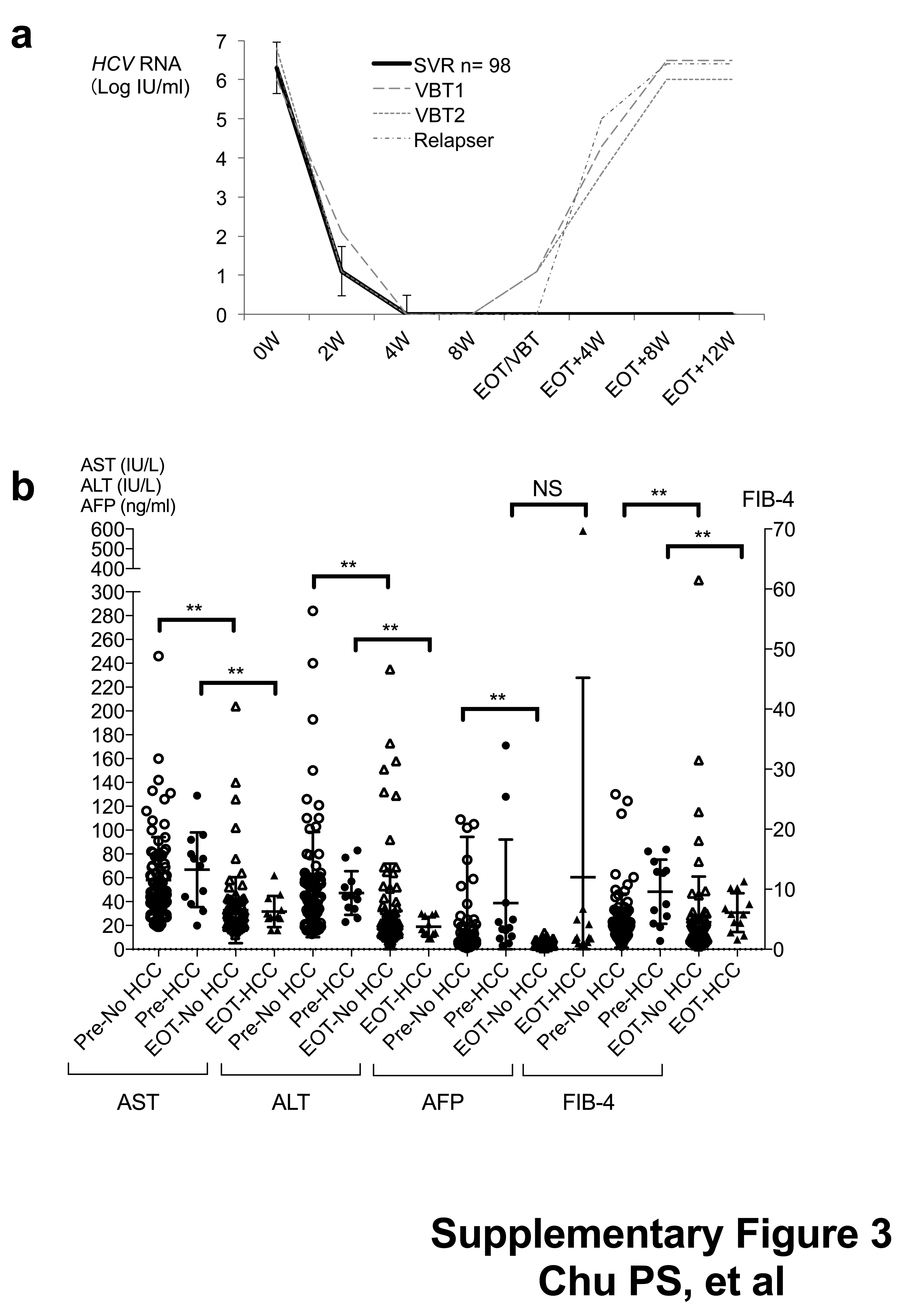

Supplement: S3 Fig — Viral dynamics (a) and biochemial changes (b) stratified by early HCC emergence in IFN−/DAA group. Statistics were shown as mean with SD. *, P< 0.05; **, P< 0.01. Units: HCV-RNA: LogIU/ml; AST: IU/L; ALT: IU/L; AFP: ng/ml. VBT, viral breakthrough. (TIFF) [file pone.0179096.s003.tiff]

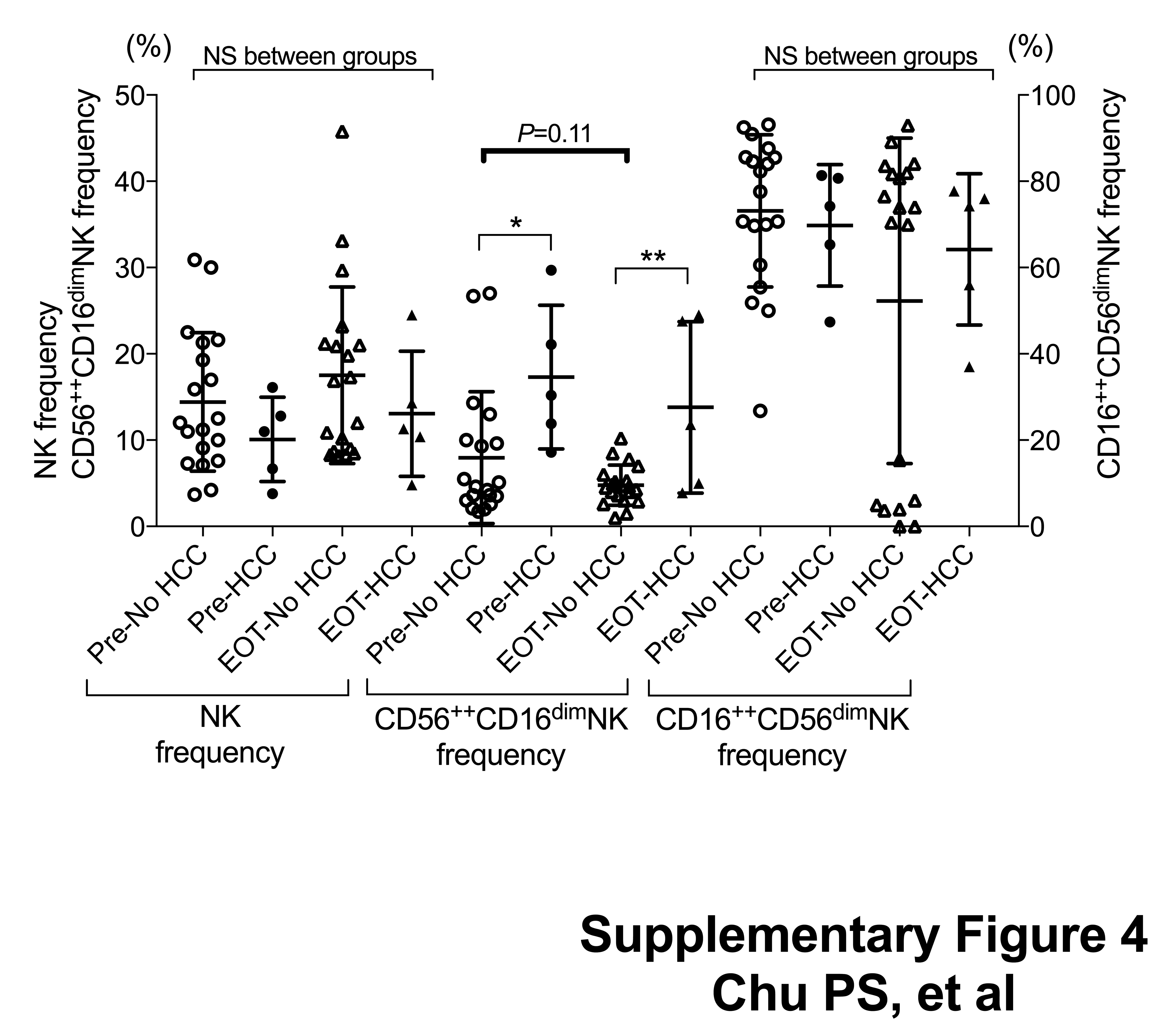

Supplement: S4 Fig — Statistics were shown as mean with SD. *, P< 0.05; **, P< 0.01. NS, not significant. (TIFF) [file pone.0179096.s004.tiff]

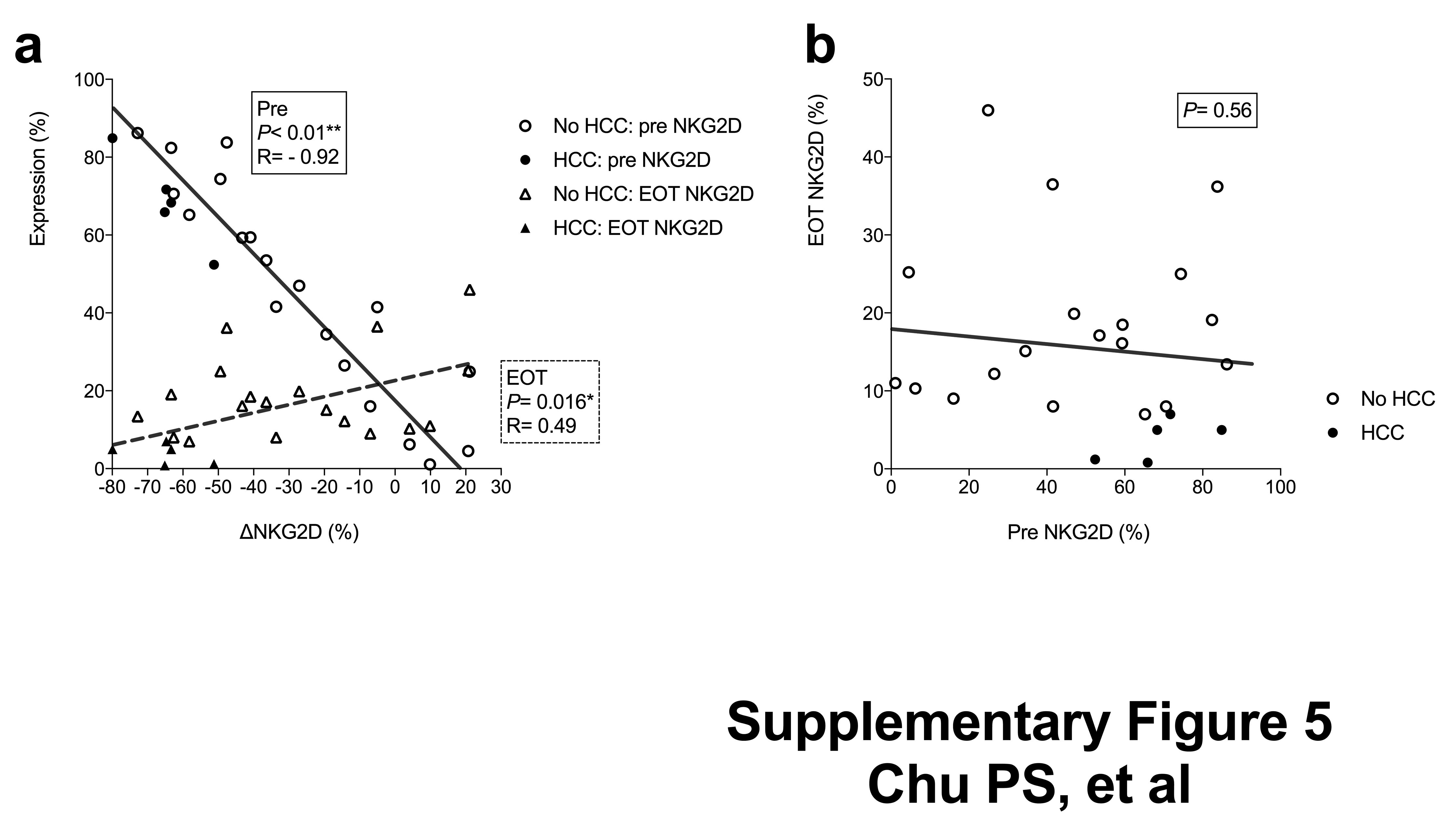

Supplement: S5 Fig — (a) Linear regression analyses of ΔNKG2D and pre- or EOT NKG2D expressions. (b) Linear regression analyses of pre- and EOT NKG2D expressions. Filled circles represent cases with early emerging HCC and open circles represent cases without. R, correlation coefficient; *, P< 0.05; **, P< 0.01. (TIFF) [file pone.0179096.s005.tiff]

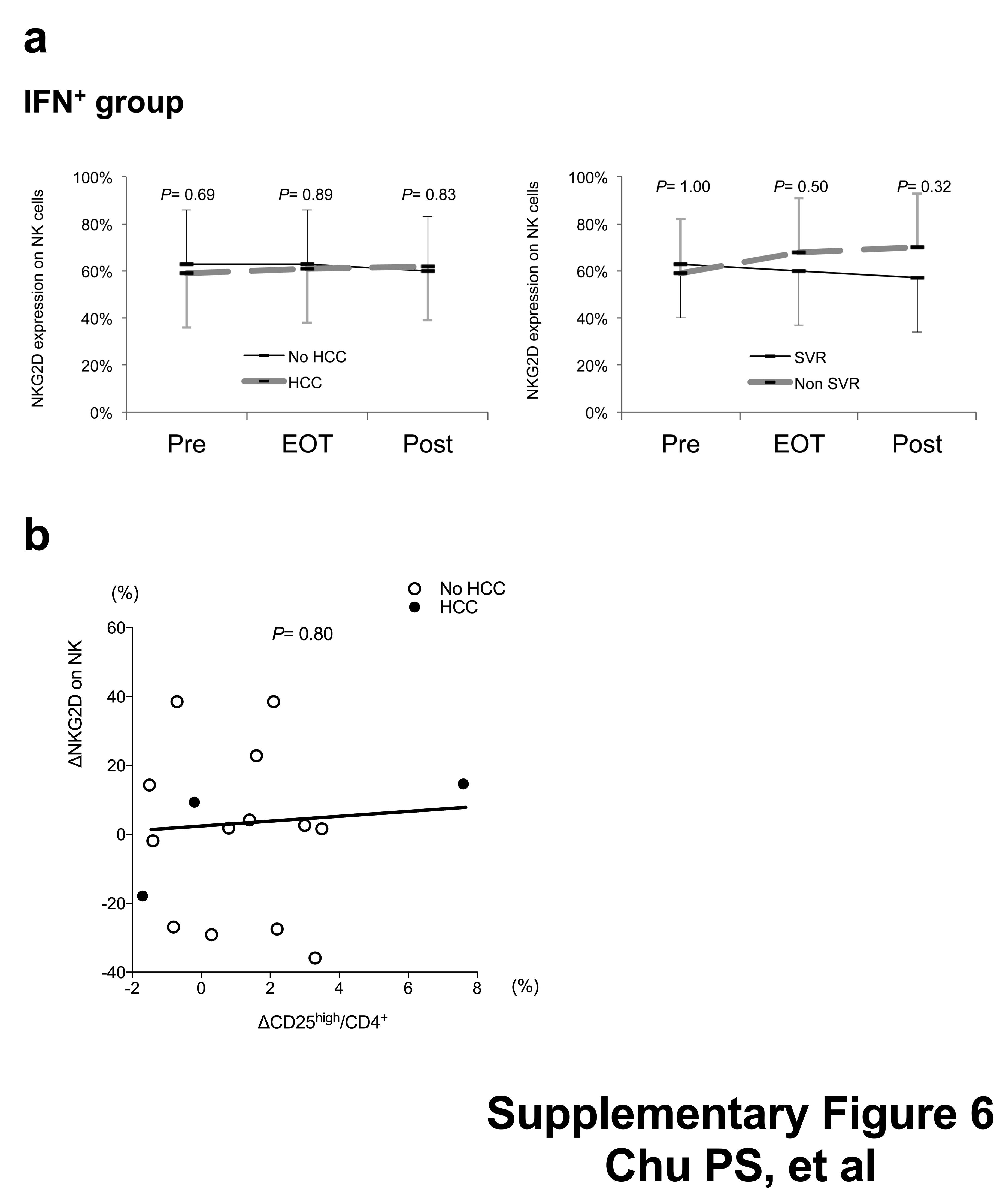

Supplement: S6 Fig — (TIFF) [file pone.0179096.s006.tiff]
