# Supplementary material for: On-treatment decrease of NKG2D correlates to early emergence of clinically evident hepatocellular carcinoma after interferon-free therapy for chronic hepatitis C
Source: PLoS One. 2017 Jun 15;12(6):e0179096. doi: 10.1371/journal.pone.0179096 (PMC5472371; doi:10.1371/journal.pone.0179096)
Supplement: S1 Table — CPT, Child-Pugh-Turcotte scores; PS, performance status; R/O, recurrence or occurrence; RFA, radiofrequency ablation. TACE, trans-arterial chemoembolization. (For other abbreviations, please see the main text.). (DOCX) [file pone.0179096.s007.docx]

S1 Table. Liver function and HCC-related variables of patients with early emerging HCC (recurrence/occurrence) at three time-points of the study.

| No. | R/O | Age | Sex | DAA | SVR | At HCC treatment (or last evaluation) before DAA | | | | | Non-recurrence duration to DAA start (months) | DAA start | | | EOT to HCC (months) | At HCC Emergence | | | | | Status at end of F/U |
| --- | --- | --- | --- | --- | --- | --- | --- | --- | --- | --- | --- | --- | --- | --- | --- | --- | --- | --- | --- | --- | --- |
|  |  |  |  |  |  | AFP | PS | CPT | BCLC Stage | HCC treatment |  | AFP | PS | CPT |  | AFP | PS | CPT | BCLC  Stage | HCC treatment |  |
| 1 | R | 77 | F | D/A | Y | 10 | 0 | 6 | A | RFA | 8 | 17 | 0 | 5 | 2 | 13 | 0 | 5 | A | RFA | Alive |
| 2 | R | 79 | F | D/A | N | 137 | 0 | 6 | A | RFA | 18 | 128 | 0 | 7 | 1 | 10 | 0 | 5 | 0 | RFA | Alive |
| 3 | R | 50 | M | D/A | Y | 80 | 0 | 7 | 0 | RFA | 27 | 23 | 0 | 6 | 4 | 12 | 0 | 5 | 0 | Hepatectomy | Alive |
| 4 | R | 58 | F | S/L | Y | 14 | 0 | 6 | 0 | RFA | 27 | 18 | 0 | 6 | 1 | 8 | 0 | 5 | 0 | RFA | Alive |
| 5 | R | 80 | F | S/L | Y | 5 | 1 | 5 | 0 | RFA | 9 | 4 | 0 | 5 | 1 | 34 | 1 | 5 | 0 | RFA | Alive |
| 6 | R | 62 | M | D/A | Y | 7 | 1 | 5 | B | TACE | 18 | 25 | 0 | 5 | 1 | 6 | 1 | 5 | B | RFA+ TACE | Alive |
| 7 | R | 82 | F | S/L | Y | 2 | 1 | 5 | B | TACE | 28 | 3 | 1 | 5 | 2 | 3 | 1 | 5 | B | TACE | Alive |
| 8 | R | 67 | F | S/L | Y | 30 | 0 | 6 | A | RFA | 3 | 39 | 0 | 6 | 3 | 9 | 0 | 5 | B | TACE | Alive |
| 9 | O | 65 | F | D/A | N |  | | | | | | 9 | 0 | 5 | 5 | 4 | 0 | 5 | 0 | RFA | Alive |
| 10 | O | 79 | F | D/A | Y |  |  |  |  |  |  | 16 | 0 | 5 | 1 | 20 | 0 | 5 | A | RFA | Alive |
| 11 | O | 73 | F | D/A | Y |  | | | | | | 171 | 0 | 5 | 2 | 591 | 0 | 5 | A | RFA | Alive |
| 12 | O | 72 | M | S/L | Y |  | | | | | | 6 | 0 | 5 | 4 | 5 | 0 | 5 | A | RFA | Alive |

CPT, Child-Pugh-Turcotte scores; PS, performance status; R/O, recurrence or occurrence; RFA, radiofrequency ablation. TACE, trans-arterial chemoembolization.

(For other abbreviations, please see the main text
