# Supplementary material for: On-treatment decrease of NKG2D correlates to early emergence of clinically evident hepatocellular carcinoma after interferon-free therapy for chronic hepatitis C
Source: PLoS One. 2017 Jun 15;12(6):e0179096. doi: 10.1371/journal.pone.0179096 (PMC5472371; doi:10.1371/journal.pone.0179096)
Supplement: S2 Table — (DOCX) [file pone.0179096.s008.docx]

S2 Table. Clinical characteristics of IFN^-^/DAA and IFN^-^/DAA-FU groups

|  | | IFN^-^/DAA | | IFN^-^/DAA-FU | | *P* |
| --- | --- | --- | --- | --- | --- | --- |
| *N* | | 101 | | 24 | | – |
| Age, years | | 67 [24–85] | | 67.5 [45–79] | | 0.69 |
| Sex (M/F), n | | 38/ 63 | | 10 /14 | | 0.82 |
| Pre-treatment |  | |  | |  |  |
| ALT, IU/L | | 44[13–284] | | 58 [26–284] | | 0.05 |
| AST, IU/L | | 49[19–246] | | 67 [27–246] | | 0.07 |
| G-GTP, IU/L | | 31[11–268] | | 43 [19–268] | | 0.05 |
| T-Bil, mg/dl | | 0.8[0.3-2.7] | | 0.9 [0.4–2.7] | | 0.17 |
| Albumin, g/dl | | 4.3[3.3–4.9] | | 3.9 [3.3–4.8] | | 0.06 |
| Platelet, 10^3^/μl | | 136[33–457] | | 108 [47–221] | | 0.07 |
| 4COL7s, ng/ml | | 6.7[3.4–17] | | 7.9 [3.9–15] | | 0.06 |
| AFP, ng/ml | | 6[1–685] | | 12 [3–171] | | 0.29 |
| *HCV*-RNA, LogIU/ml | | 6.3[3.5–7.5] | | 6.3 [5.8–7.5] | | 0.47 |
| Total cholesterol, mg/dl | | 159[107–241] | | 148 [107–230] | | 0.23 |
| FIB-4 | | 3.87 [0.62–107] | | 4.49 [1.21–25.80] | | 0.12 |
| APRI | | 1.11[0.13–52] | | 1.80[0.35–14.95] | | 0.07 |
| Previous HCC, *n* (%) | | 16 (16) | | 5 (21) | | 0.55 |
| NKG2D, % | |  | |  | | 0.61 |
| Post- treatment |  | |  |  |  |  |
| EVR, *n* (%) | | 86 (87) | | 20 (83) | | 0.74 |
| SVR, *n* (%) | | 98 (97) | | 21 (88) | | 0.08 |
| Early HCC, *n* (%) | | 12 (12) | | 5 (21) | | 0.52 |
