# Supplementary material for: On-treatment decrease of NKG2D correlates to early emergence of clinically evident hepatocellular carcinoma after interferon-free therapy for chronic hepatitis C
Source: PLoS One. 2017 Jun 15;12(6):e0179096. doi: 10.1371/journal.pone.0179096 (PMC5472371; doi:10.1371/journal.pone.0179096)
Supplement: S3 Table — (DOCX) [file pone.0179096.s009.docx]

S3 Table. Clinical characteristics of IFN^-^/DAA-FU and IFN^+^ groups

| Variables | IFN^-^/DAA-FU | IFN^+^ | *P* |
| --- | --- | --- | --- |
| *N* | 24 | 16 | – |
| Age, years | 67.5 [45–79] | 63.5 [47–83] | 0.61 |
| Sex (M/F), *n* (%) | 10 (42)/14 (58) | 5 (31)/11 (69) | 0.52 |
| Pre-treatment parameters | | | |
| ALT, IU/L | 58 [26–284] | 69 [18–197] | 0.68 |
| Albumin g/dl | 3.9 [3.3–4.8] | 3.9 [3.2–4.9] | 0.68 |
| Platelets, 10^3^/mcl | 108 [47–221] | 162 [94–296] | <0.01** |
| 4COL7s, ng/ml | 7.9 [3.9–15] | 5.7 [4.2–11] | 0.02* |
| AFP, ng/ml | 12 [3–171] | 6.5 [1–30] | 0.047* |
| *HCV*-RNA, Log IU/ml | 6.3 [5.8–7.5] | 6.75 [5.7–7.7] | 0.02* |
| Total cholesterol, mg/dl | 148 [107–230] | 188.5 [108–230] | 0.02* |
| Previous HCC, *n* (%) | 5 (21) | 2 (13) | 0.68 |
| Pre-treatment NKG2D expression on NK cells, % | 0.56 [0.01–0.86] | 0.61 [0.28–0.86] | 0.29 |
| Post-treatment parameters | | | |
| SVR, *n* (%) | 21 (88) | 11 (69) | 0.23 |
| Duration from EOT to HCC (months) | 2 [1–5] | 12 [2–18] | <0.01 |
| HCC emergence during F/U, *n* (%) | 5 (21) | 3 (19) | 1.00 |
